# Total white blood cell count or neutrophil count predict ischemic stroke events among adult Taiwanese: report from a community-based cohort study

**DOI:** 10.1186/1471-2377-13-7

**Published:** 2013-01-15

**Authors:** Tzy-Haw Wu, Kuo-Liong Chien, Hung-Ju Lin, Hsiu-Ching Hsu, Ta-Chen Su, Ming-Fong Chen, Yuan-Teh Lee

**Affiliations:** 1 Department of Internal Medicine, National Taiwan University Hospital, Taipei, 100, Taiwan; 2 Institute of Epidemiology & Preventive Medicine, National Taiwan University, Taipei, Taiwan; 3 China Medical University Hospital, Taichung, Taiwan

**Keywords:** White blood cell count, Neutrophil count, Ischemic stroke event

## Abstract

**Background:**

Evidence about whether white blood cell (WBC) or its subtypes can act as a biomarker to predict the ischemic stroke events in the general population is scanty, particularly in Asian populations. The aim of this study is to establish the predictive ability of total WBC count or subtypes for long-term ischemic stroke events in the cohort population in Taiwan.

**Methods:**

The Chin-Shan Community Cohort Study began from 1990 to 2007 by recruiting 1782 men and 1814 women of Chinese ethnicity. Following a total of 3416 participants free from ischemic stroke events at baseline for a median of 15.9 years; we documented 187 new incident cases.

**Results:**

The multivariate relative risk for the comparison of the participants in the fifth and first WBC count quintiles was 1.67 (95% confidence interval [CI], 1.02–2.73; *P* for trend=0.03), and the corresponding relative risk for neutrophil count was 1.93 (95% CI, 1.13–3.29; *P* for trend=0.02). The discriminative ability by WBC and neutrophil counts were similar (area under the receiver operating characteristic curve, 0.600 for adding WBC, 0.610 for adding neutrophils, 0.595 for traditional risk factor model). In addition, the net reclassification improvement (NRI) values between the neutrophil and white blood cell count models were not significant (NRI, =-2.60%, *P=*0.35), indicating the similar discrimination performance for both WBC and neutrophil counts.

**Conclusions:**

WBC and neutrophil count had a similar ability to predict the long-term ischemic stroke events among Taiwanese.

## Background

Total white blood cell (WBC) count is an indicator for acute or chronic inflammation, and an elevated WBC count also is a risk factor for atherosclerotic vascular disease. WBC derived a series of phagocyte reactions believed to contribute to vascular injury and atherosclerotic progression [1-3]. Several prospective studies have shown a positive and independent association between WBC count and risk for coronary heart disease (CHD) [3,4], stroke [3,5], and all-cause mortality [6,7]. Furthermore, the subtypes of WBC are also known to be biomarkers for cardiovascular or stroke risk prediction. Some studies found that leukocyte count is associated with aortic arch plaque thickness [8], progression of aortic atheroma in patients with stroke [9], or increased risk of stroke and vascular death in patients with symptomatic intracranial atherosclerotic disease [10]. Other studies have showed that neutrophil count adds prognostic information to major adverse cardiac events in acute coronary syndrome [11] or as an independent predictor of recurrent ischemic stroke [12]. In addition, one study demonstrated that granulocyte count was the strongest biomarker for the association between CHD, ischemic stroke incidence and cardiovascular disease mortality [3].

However, most evidence has come from studies of western populations and few data are available on predicting ischemic stroke events in eastern people, leaving uncertainty as to the generalized ability of the findings. Moreover, it is still not clear that whether WBC or its subtypes are the best biomarker to predict the ischemic stroke events in the general population, particularly among Asian people. Therefore, the aim of this study is to establish the predictive ability of total WBC count or subtypes for long-term ischemic stroke events in a cohort population in Taiwan.

## Methods

### Study design and participants

Details of this cohort study have been published previously [13-15]. Briefly, the Chin-Shan Community Cohort Study began in 1990 by recruiting 1782 men and 1814 women of Chinese ethnicity from the town of Chin-Shan, 30 kilometers north of Taipei, Taiwan. Information about lifestyle and medical conditions and anthropometric measures was assessed by interview questionnaires and physical examinations; the validity and reliability of the collected data and measurements have been reported in detail [15,16]. The procedure of follow-up strategy and outcome ascertainment for our documentation of incident ischemic stroke have been previously described and validated [13,17]. For the establishment of the predictive ability of total WBC count or subtypes for long-term ischemic stroke events in this study, participants who had a previous history of stroke (n=89) were excluded from this study. We recruited incident stroke cases during 1990-2007. The incident of new stroke cases was ascertained according to the following criteria: a sudden neurological symptom of vascular origin that lasted > 24 hours with supporting evidence from an image study; fatal stroke cases were included. The transient ischemic attacks and hemorrhagic stroke were excluded in this study because the former is difficult to define and the latter has different pathogenetic mechanism from ischemic stroke. The cases were confirmed by cardiologists and neurologists, and the National Taiwan University Hospital (NTUH) Committee Review Board approved the study protocol. Participants with incomplete blood data (n=40) at baseline were also excluded from this investigation. After the exclusion of previous history of stroke, transient ischemic attacks, hemorrhagic stroke and incomplete blood data cases, the final analytic sample included 3416 participants.

The follow-up and verification for events and deaths were reviewed by physicians on the Committee of Mortality and Morbidity within the study team. Medical records were used to determine the possibility cause of death. Four cardiologists were responsible for reviewing the questionnaires, medical records, and laboratory reports to determine whether each event met the protocol established by the project steering committee, and addition to household visits to verify the causes of deaths and events. Relatives of nonrespondents were contacted to obtain information on the health status of uncooperative individuals. Medical records were reviewed at Chin-Shan Community Health Center, local clinics, and NTUH. By using national vital statistics records, the study team was able to obtain the information about the deceased number of participants and determine the attributed cause of death.

The informed consent was given for all participants in this study and all of our enrolled participants agreed.

### Measurement of clinical, lifestyle, and biochemical markers

The procedures for clinical and biochemical measures have been reported elsewhere [15,16]. In short, blood pressure was measured twice in the right arm using a mercury sphygmomanometer with the subject seated comfortably and arms supported and positioned at the level of the heart.

The average of the blood pressure measurements was used as described previously [14,18]. All venous blood samples drawn after a 12-hour overnight fast were immediately refrigerated and transported within 6 hours to the NTUH. Serum samples were then stored at -70°C before batch assay for levels of total cholesterol, triglyceride, and high-density lipoprotein cholesterol. Standard enzymatic tests for serum cholesterol were used (Merck 14354 and 14366, respectively). Blood samples for glucose analysis were drawn into glass test tubes each containing 80 mol/L fluoride/oxalate reagents after centrifugation by 1500 *g* for 10 minutes; glucose levels were measured in supernatant by enzymatic assay (Merck 3389 commercial kit) in an Eppendorf 5060 autoanalyzer. The peripheral blood cell analysis was measured using a blood cell counter (Sysmex Cell Counter NE-8000; TOA Medical Electronics Co Ltd, Kobe, Japan).

### Statistical analysis

We classified participants on the basis of quintiles of white blood cell count, and continuous variables were presented as the mean (SD) or as the median.

Relationships between the baseline white blood cell count and other biomarkers were examined by evaluating age- and sex-adjusted Spearman partial correlation coefficients. The incidence rates of ischemic stroke were calculated by dividing the number of cases by the number of person-years of follow-up for each white blood cell count quintile. The hazard ratio (HR) of ischemic stroke was calculated by dividing the incidence rate for each quintile by the rate in the first quintile. We used Cox proportional hazards models to adjust for potential confounding variables, including age group (35-44, 45-54, 55-64, 65-74, or ≧ 75 years), sex, BMI (< 18, 18-20.9, 21-22.9, 23-24.9, or ≧ 25 kg/m^2^), alcohol intake, smoking, regular exercise, marital status, educational level (< 9/ ≧ 9 years) , occupation (no work, manual work, or professional), family history of diabetes and hypertension, regular exercise, history of diabetes, hypertension, coronary heart disease, atrial fibrillation, and left ventricular hypertrophy. To test for a linear trend across white blood cell count quintiles, we used the median white blood cell count for each category as a continuous variable in the multivariate model. We also used the test of Hosmer and Lemeshow to evaluate the goodness of fit for the model [19] and the statistics were not rejected. In addition, we provided several additional statistics, including net reclassification improvement (NRI) and integrated discrimination improvement (IDI) for the comparison between white blood cell count and neutrophil count models [20] because the areas under the curve (AUC) value are not the best discriminatory statistics for prediction power.

The NRI statistic was based on the reclassification tables and was calculated from a sum of differences between the ‘upward’ movement in categories for event participants and the ‘downward’ movement in those for nonevent participants. We presented the NRI according to the quintile of WBC and neutrophil count. The IDI can be interpreted as a difference between improvement in average sensitivity and any potential increase in average ‘1–specificity’ and the statistic was a difference in Yates discrimination slopes between the new and old models. All statistical tests were 2-tailed and *P* values less than 0.05 were considered statistically significant. Analyses were performed with SAS software (version 9.1; SAS Institute) and Stata (version 9.1; Stata Corporation).

## Results

Participants in the highest WBC count quintile were more likely to be male and more likely to smoke and drink alcohol than the participants in the other quintiles. They also had a higher prevalence of the history of hypertension, diabetic mellitus, BMI, systolic and diastolic blood pressure, higher concentration of cholesterol, triglycerides, HDL and LDL cholesterol (Table 1). For the cohort follow-up from 1990 to the end of 2007 (median 15.9 years, interquintiles range: 12.8 to 16.9 years), we documented 187 new incident cases of ischemic stroke (including cases of ischemic and undetermined type). Table 2 shows the HRs and 95% confidence intervals (CIs) for the WBC count quintile at baseline. After adjustment for age, sex, lifestyle, the HRs according to white blood cell count quintile were 1.25, 1.19, 1.65, and 2.06 (95% CI, 1.30 –3.27; *P* for trend = 0.0006). After additional adjustment for the other covariates, the HR for the comparison of the participants in the fifth and first WBC count quintiles was 1.67 (95% CI, 1.02–2.73; *P* for trend = 0.03). Table 3 shows the HRs and 95% confidence intervals (CIs) for neutrophil count quintile at baseline. After adjustment for age, sex, lifestyle, the HRs according to neutrophil count quintile were 1.63, 1.47, 1.72, and 2.32 (95% CI, 1.39 –3.87; *P* for trend =0.002). After additional adjustment for the other covariates, the HR for the comparison of the participants in the fifth and first neutrophil count quintiles was 1.93 (95% CI, 1.13–3.29; *P* for trend=0.02). For the lymphocyte counts, the association was not significant (data not shown).

**Table 1:** Baseline characteristics by quintiles of white blood cell count in 3416 individuals

	**Quintile of baseline WBC count**					
	**1**	**2**	**3**	**4**	**5**	**p**
participants, n	715	715	672	673	641	
Gender						<.0001
men, %	36.8	43.9	49.3	49.5	56.9	
women, %	63.2	56.2	50.6	50.7	43.5	<.0001
Current smoker (yes), %	26.6	30.8	39.3	38.2	48.0	<.0001
Alcohol drinking (yes), %	25.2	27	32.7	30.6	33.2	0.002
Marital status						0.3
single, %	2.2	2.7	3.1	3.9	2.3	
Live with spouse, %	86.2	85.6	85.9	87.7	85.5	
Divorced or separated, %	11.6	11.6	11.1	8.5	12.0	
Education level						0.004
<9 years, %	96.6	95	93.8	92.1	93.1	
≧9 years, %	3.4	5.5	6.3	7.9	6.9	
Job status						0.04
No job, %	53.6	50.6	48.8	47	44.8	
Farmer, laborer, %	32.0	35.7	34.1	35.1	37.4	
Professional, business, %	14.4	13.7	17.1	18	17.8	
Regular exercise (yes), %	17.2	14.5	16.1	13.7	12.8	0.14
History of hypertension, %	25.3	23.9	29.5	31.8	30.4	0.005
History of diabetic mellitus, %	8.7	10.5	11.8	15.9	17.9	<.0001
Age, years*	55.3	54.8	54.4	53.6	54.6	0.1647
BMI, kg/m^2^*	22.6	23.2	23.5	24.1	24.0	<.0001
Systolic blood pressure, mmHg*	123	122	126	126	127	<.0001
Diastolic blood pressure, mmHg*	76	76	77	78	78	<.0001
Total cholesterol, mg/dL*	190.3	193.4	200.8	199.6	205.3	<.0001
Triglycerides, mg/dL*	99.7	110.8	131.5	133.9	155.0	<.0001
HDL cholesterol, mg/dL*	50.1	48.9	46.7	46.2	45.7	<.0001
LDL cholesterol, mg/dL*	128.8	132.8	140.7	140.4	145.9	<.0001
Coronary heart disease, %	4.6	4.8	4.8	5.1	4.8	1
Atrial fibrillation, %	1	1.1	1.3	0.7	0.9	0.86
Left ventricular hypertrophy, %	6.4	7.1	7.7	7.9	9.4	0.34

* Data are expressed as the mean.

**Table 2:** Median WBC count, number of study participants, incidence case, person-years, rate, and HRs by WBC quintile for the association with ischemic stroke in the participants

	**Quintile of baseline white blood cell count**						
	**Q1**	**Q2**	**Q3**	**Q4**	**Q5**	**P for trend**	**P for Hosmer and Lemeshow test**
Median WBC count, per μl	4400	5400	6100	7000	8500		
Individuals, n	715	715	672	673	641		
Incident cases, n	31	35	32	42	47		
person-years	10232	10094	9548	9568	8731		
Rate(/1000 person-year)	3	3.5	3.4	4.4	5.4		
HR, model 1*^,†^	1	1.21 (0.74-1.96)	1.17 (0.71-1.91)	1.62 (1.02-2.58)	2.00 (1.27-3.15)	0.0008	0.72
HR, model 2*^,‡^	1	1.25 (0.76-2.03)	1.19 (0.72-1.97)	1.65 (1.03-2.64)	2.06 (1.30-3.27)	0.0006	0.33
HR, model 3*^,§^	1	1.18 (0.72-1.95)	1.00 (0.59-1.67)	1.34 (0.82-2.19)	1.67 (1.02-2.73)	0.03	0.23

*Data for HR (95% CI) are expressed relative to WBC count quintile1.

^†^Model 1: Adjusted for age and sex.

^‡^Model 2: Model 1 plus BMI, alcohol intake, smoking, marital status, education level, occupation, exercise, family history of diabetes and hypertension.

^§^Model 3: Model 2 plus systolic blood pressure, diastolic blood pressure, diabetes, total cholesterol, triglyceride, low density lipoprotein, high density lipoprotein, atrial fibrillation, left ventricular hypertrophy, and coronary artery disease.

**Table 3:** Median neutrophil count, number of study participants, incidence case, person-years, rate, and HRs by neutrophil quintile for the association with ischemic stroke in the participants

	**Quintile of baseline neutrophil count**						
	**Q1**	**Q2**	**Q3**	**Q4**	**Q5**	**P for trend**	**P for Hosmer and Lemeshow test**
Median neutrophil count, per μl	2500	3300	3800	4500	5900		
Individuals, n	694	615	603	631	610		
Incident cases, n	24	35	28	37	44		
person-years	10113	8756	8476	8939	8238		
Rate(/1000 person-year)	2.4	4	3.3	4.1	5.3		
HR, model 1*^,†^	1	1.59 (0.95-2.68)	1.41 (0.82-2.43)	1.66 (0.99-2.79)	2.27 (1.38-3.76)	0.002	0.79
HR, model 2*^,‡^	1	1.63 (0.96-2.77)	1.47 (0.85-2.56)	1.72 (1.02-2.91)	2.32 (1.39-3.87)	0.002	0.25
HR, model 3*^,§^	1	1.52 (0.88-2.61)	1.22 (0.69-2.17)	1.52 (0.89-2.62)	1.93 (1.13-3.29)	0.02	0.45

*Data for HR (95% CI) are expressed relative to WBC count quintile1.

^†^Model 1: Adjusted for age and sex.

^‡^Model 2: Model 1 plus BMI, alcohol intake, smoking, marital status, education level, occupation, exercise, family history of diabetes and hypertension.

^§^Model 3: Model 2 plus systolic blood pressure, diastolic blood pressure, diabetes, total cholesterol, triglyceride, low density lipoprotein, high density lipoprotein, atrial fibrillation, left ventricular hypertrophy, and coronary artery disease.

**Figure 1:**
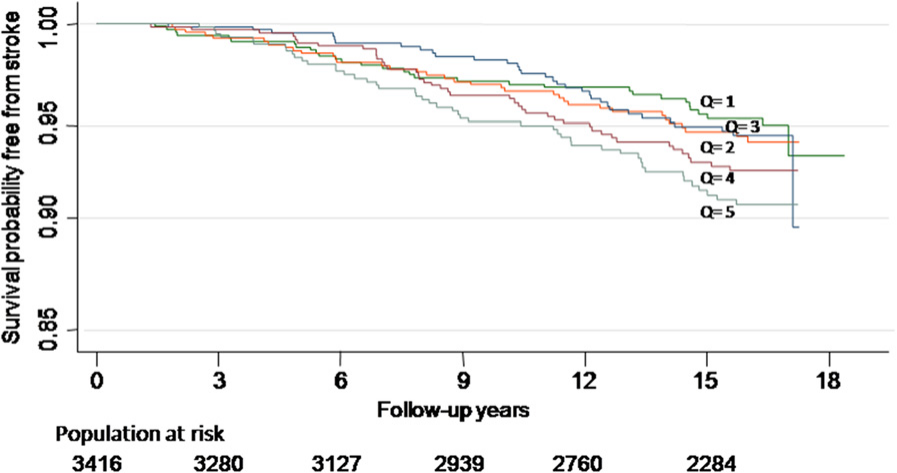
**Kaplan–Meier survival estimates of ischemic stroke events by five different WBC count quintiles in the Chin-Shan Community cohort study with a total of 3416 participants, and were followed up for 17 years.** The bottom numbers showed patients remaining at each time period.

The Kaplan–Meier survival estimates of ischemic stroke events associated with each quintile of leukocyte count in participants are shown in the Figure 1. The logrank test showed that the survival curves were significantly different (*P* < 0.01).

**Figure 2:**
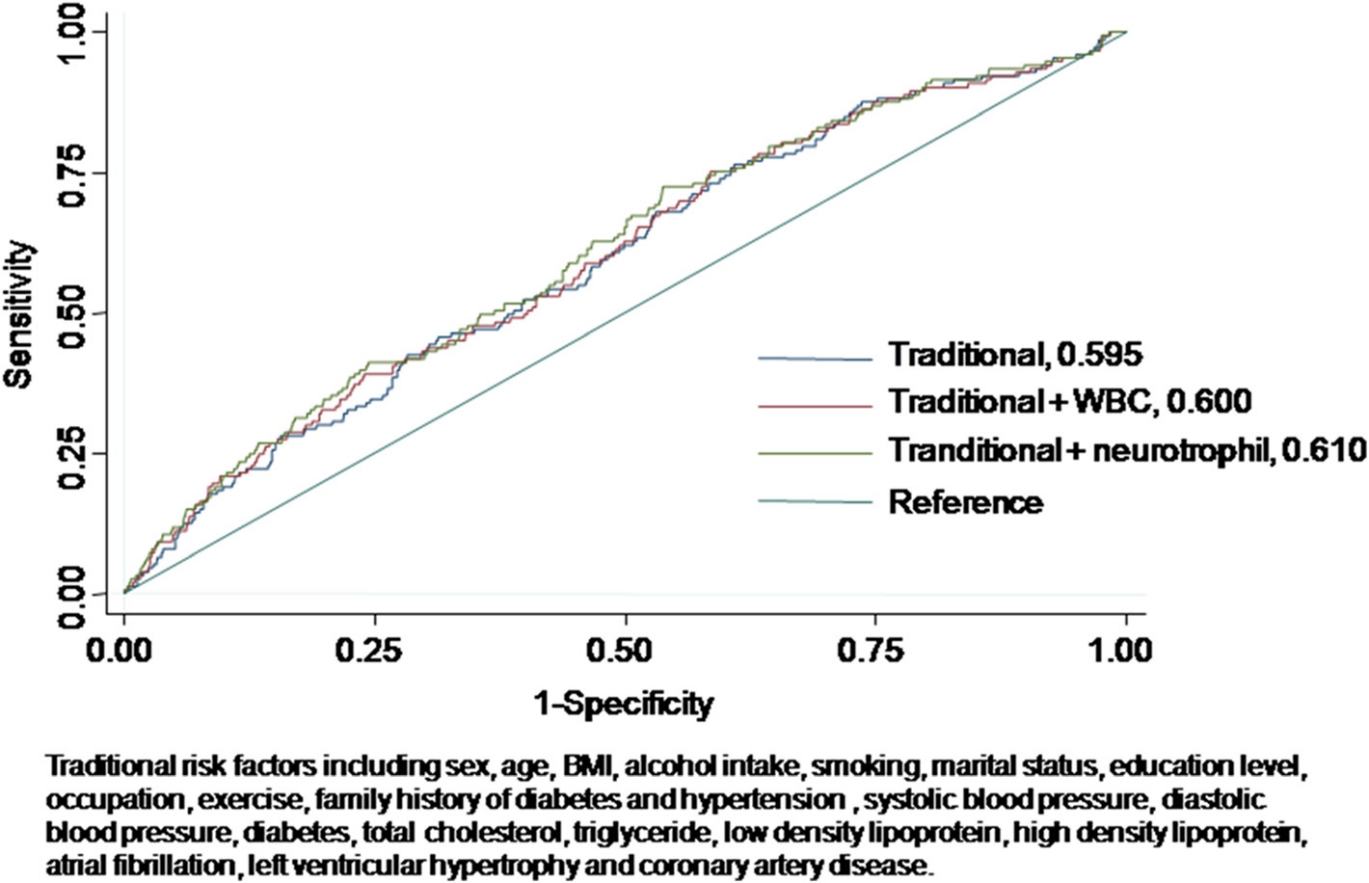
**Areas under ROC curve comparison for ischemic stroke events in the Chin-Shan Community cohort study with a total of 3416 participants.** The discriminative ability by WBC and neutrophil counts was similar, that is AUC readings were 0.595 for traditional risk factor model, 0.600 for the addition with WBC, 0.610 for the addition with neutrophils. The traditional risk factors were shown at the bottom of this figure.

Predictive ability was tested by the following performance measures (Figure 2). First, the discriminative ability by WBC and neutrophil counts was similar (the AUC, 0.600 for adding WBC, 0.610 for adding neutrophils, 0.595 for traditional risk factor model). Second, the NRI and IDI values between the traditional and white blood cell count models were not significant (NRI =2.1%, *P=*0.36; IDI= 0.003, *P=*0.01), the NRI and IDI values between the traditional and neutrophil models were not significant (NRI =-0.92%, *P=*0.77; IDI= 0.007, *P<*0.01),and the NRI and IDI values between the white blood cell count and neutrophil models were not significant (NRI =-2.60%, *P=*0.35; IDI= 0.03, *P=*0.02), indicating a similar discrimination performance between the neutrophil count and WBC count model for predicting the risk of long term ischemic stroke events. Higher WBC count, as shown in Table 2, predisposed the subjects to have a higher risk of developing stroke with an increasing significant trend across the WBC quintile groups (all three multivariable-adjusted models, p<0.05 in trend). Similarly, increased neutrophil count was associated with raised risk for occurrence of stroke, after clinical confounding factors were adjusted (in Table 3, p<0.05 in trend). As compared with the traditional prediction model, including WBC or neutrophil count into the traditional stroke prediction model, moreover, raised the general discriminatory abilities to identify subjects at greater risk of stroke (Table 4).

**Table 4:** Comparison of general discriminating performance between neutrophil-included and WBC-included models

	**C-statistic**			**Integrated discrimination improvement (IDI)**		
		**95% CI**	**p**	**IDI**	**95% CI**	**p-value**
Traditional model	0.59	0.55 to 0.64		reference		
vs						
WBC-included model	0.60	0.55 to 0.65	0.18	0.003	0.0004 to 0.006	0.01
Traditional model	0.59	0.55 to 0.64		reference		
vs						
Neutrophil-included model	0.61	0.56 to 0.66	0.002	0.007	0.004 to 0.01	<0.01

## Discussion

This prospective cohort study based on ethnic Chinese in Taiwan discovered that both WBC count and neutrophil count could predict future ischemic stroke events. The prediction was still significant after adjusting multiple ischemic stroke risk factors. Overall, there were a 67% and 93% increase in risk of ischemic stroke compared with the WBC count and neutrophil count for each first and fifth quintile respectively. We also examined the lymphocyte count for predictive ability of ischemic stroke events in this study but no difference was found between different quintiles. A similar finding was noted by a Northern Manhattan study, which demonstrated that relative elevation in WBC count predicts first cerebral infarction, and is also associated with myocardial infarction and vascular death [21]. However, the researchers did not mention the effect of ischemic stroke associated with WBC differentials because they had no data. Another study has found that elevated WBC count is directly associated with the risk of CHD and ischemic stroke incidence and mortality from cardiovascular disease [3]. After further examining the association between differential WBC counts, these researchers showed that granulocyte and monocyte counts have a strong association with ischemic stroke incidence but the same results were not found after analysis of lymphocytes. However, the study’s focus was solely on the population group of African-Americans and Caucasian. In an Asian population study, researchers observed a positive association between increased WBC count and risk of ischemic stroke but the relationship between WBC count and the risk of hemorrhagic stroke was not significant [22]. These results are also found in our data, but this study did not discuss the relationship between ischemic stroke and WBC subtypes. Another study found that WBC counts mainly neutrophil counts was independently associated with recurrent ischemic events in high risk populations [12]. Although those data did not all yield the same results, that the leukocyte and its subtypes could increase the risk of ischemic stroke can’t be denied.

The reason why elevated leukocyte counts or its subtypes increased cardiovascular and ischemic stroke risk is still not fully understood. Several potential mechanisms have nonetheless been proposed. Elevated WBC count may enhance atherogenesis and is also reflected in the inflammatory activity of atherosclerosis that causes vascular injury and tissue ischemia [1]. Neutrophil plays a key role in plaque development and instability [11], Neutrophil also releases autacoids, which induce vasoconstriction and platelet aggregation [23]. Monocytes are believed to be involved in the pathogenesis of atherosclerosis and present in every phase of atherogenesis [11]. Monocyte-derived macrophages produce cytokine, or other oxidants that can induce endothelial cell injury and succeeding thrombus formation [2,24]. Furthermore, lymphocytes play a role in chronic inflammation, and the circulating levels of specific lymphocytes, such as the subset of T lymphocytes, may be associated with unstable angina and recurrent ischemic stroke [25,26]. Although WBC and subtypes can lead to vascular injury and subsequent ischemic stroke events, we still don’t know which one is the strongest predictor for ischemic stroke events in the long term follow up.

To indentify which one is the strongest biomarker for predicting ischemic stroke events in the long term follow up, we used NRI and IDI to improve the performance measures of the prediction power of adding new biomarkers. These statistics have been widely applied in evaluating the performance of prediction models and novel biomarkers [16,27].

Our study still has several potential limitations. First, we have no data about the subtypes of ischemic stroke, such as large vessel, lacunar or cardiogenic strokes. While previous studies revealed some association of leukocyte with large vessel atherosclerosis [8-10] and cardiogenic stroke [21], there was admittedly still no consistent evidence to prove the association of leukocytes with lacunar stroke, though atherosclerosis may be play a role in lacunar stroke [28]. We need to focus on this problem in further research. Second, we did not provide the monocyte information in this study. Prospective studies suggest that monocytes were associated with the ischemic stroke incidence [3] and predicted clinical course and prognosis in human stroke [29]. Lack of information may reduce power for the prediction. Third, we did not have other important inflammatory markers like C-reactive protein (CRP) and cytokines in our first blood sampling. Previous literatures reveled CRP could increase the risk of stroke and post stroke vascular disease recurrence [30,31]. Future studies should assess other markers of inflammatory activity for analysis. Finally, we tried to establish the predictive ability of WBC and neutrophil count for long-term ischemic stroke events in this study, however, the predictors chosen by us in this paper could be influenced by other environmental elements or other diseases encountered during follow-up, especially for such a long follow-up period. We may need to focus more on this limitation for our further study design and recruitment.

## Conclusion

In conclusion, the results of our prospective study indicate a positive association between the WBC, neutrophil count and long term ischemic stroke events. The WBC and neutrophil count had a similar ability to predict the long term ischemic stroke events among Taiwanese. We suggest that both WBC count and neutrophil count are considered as risk factors for the risk of long term ischemic stroke events.

## Competing interests

All authors declare that they have no competing interests.

## Authors’ contributions

THW carried out the design and data collection and drafted the manuscript. KLC carried out statistical analysis and revised the draft. HJL participated in collecting data and statistical analysis. HCH participated in laboratory data quality control and biochemical analysis. TCS participated in collecting data and maintaining the study. MFC and YTL conceived of the study, and participated in its design and coordination and helped to draft the manuscript. All authors read and approved the final manuscript.

## Pre-publication history

The pre-publication history for this paper can be accessed here:

http://www.biomedcentral.com/1471-2377/13/7/prepub
